# Supplementary material for: Land use and land cover changes along the China-Myanmar Oil and Gas pipelines – Monitoring infrastructure development in remote conflict-prone regions
Source: PLoS One. 2020 Aug 19;15(8):e0237806. doi: 10.1371/journal.pone.0237806 (PMC7437919; doi:10.1371/journal.pone.0237806)
Supplement: S1 File — (DOCX) [file pone.0237806.s001.docx]

**S1. Landsat Scenes.** Landsat scenes and tiles used in the analysis.

|  | Pipeline Route Classification | | |
| --- | --- | --- | --- |
| Tile | 2005  (Landsat 5) | 2010  (Landsat 5) | 2012  (Landsat 8) |
| 132_44 | LT51320442005061BKT02 | LT51320442010043BKT00 | LC81320442012073LGN01 |
| 133_45 | LT51330452005036BKT00 | LT51330452010043BKT00 | LC81330452012080LGN01 |
| 134_46 | LT51340462005043BKT00 | LT51340462010057BKT00 | LC81340462012071LGN01 |
| 134_47 | LT51340472005043BKT00 | LT51340472010041BKT00 | LC81340472012071LGN01 |
