# Supplementary material for: Land use and land cover changes along the China-Myanmar Oil and Gas pipelines – Monitoring infrastructure development in remote conflict-prone regions
Source: PLoS One. 2020 Aug 19;15(8):e0237806. doi: 10.1371/journal.pone.0237806 (PMC7437919; doi:10.1371/journal.pone.0237806)
Supplement: S2 File — (DOCX) [file pone.0237806.s002.docx]

**S2 File. R Scripts for Random Forest.**

The following is the R script we used to classify GeoEye1 and WorldView2 images using Random Forest.

#############################################################################

# import image

img <- brick(".tif")

img

ndvi1 <- raster("ndvi.tif")

# change band namesb

names(img) <- paste0("B", 1:3)

img

# if needed reproject from geographic coordinates to a projected coordinate reference system (CRS)

# UTM 46 N : epsg 32246

img <- projectRaster(img, crs = CRS("+init=epsg:32246"), method = "ngb") # use res argument to set output resolution

img

# examine values distribution

hist(img)

# plot image

?plotRGB

img # note min, max values in each band

plotRGB(img, r = 3, g = 2, b = 1, scale = 255)

plotRGB(img, r = 3, g = 2, b = 1) # same as above as values range between 0-255

# import shapefile

# if CRS of shapefile is not the same as img, use spTransform() to reproject it

trainData <- shapefile(".shp")

trainData

trainData$class <- data.matrix(unclass(as.factor(trainData$Name)))

# check conversion categories to numeric

for(x in unique(trainData@data$class)){

res <- subset(trainData@data, subset = class == x)

cat(paste(x, unique(res$Name), "\n"))

}

# assign name of class column to the responseCol object

responseCol <- "class"

# plot image and overlay trainData

plot(img[[1]])

plot(trainData, add = TRUE)

# if additional bands will be processed and included in img

# add them before extracting training data

nir <- raster(img, 3)

red <- raster(img, 2)

ndvi <- (nir - red)/(nir + red)

img <- brick(stack(img, ndvi))

names(img)[4] <- "ndvi" # update names

# extract training data from img using trainData polygons

# 50531 records ~ less than 0.5 min

dfAll = data.frame(matrix(vector(), nrow = 0, ncol = length(names(img)) + 1))

for (i in 1:length(unique(trainData[[responseCol]]))){

category <- unique(trainData[[responseCol]])[i]

categorymap <- trainData[c(trainData[[responseCol]] == category),]

dataSet <- extract(img, categorymap)

if(is(trainData, "SpatialPointsDataFrame")){

dataSet <- cbind(dataSet, class = as.numeric(rep(category, nrow(dataSet))))

dfAll <- rbind(dfAll, dataSet[complete.cases(dataSet),])

}

if(is(trainData, "SpatialPolygonsDataFrame")){

dataSet <- dataSet[!unlist(lapply(dataSet, is.null))]

dataSet <- lapply(dataSet, function(x){cbind(x, class = as.numeric(rep(category, nrow(x))))})

df <- do.call("rbind", dataSet)

dfAll <- rbind(dfAll, df)

}

}

# save dfAll object to recover it next session

save(dfAll, file = ".RData")

# explore data

table(dfAll$class)/nrow(dfAll)* 100

# calculate statistics for pixel values in each band

lapply(1:6, function(x){

summary(subset(dfAll, class == x))

})

# plot

plot(dfAll$B1, dfAll$B2, col = dfAll$class, asp = 1, pch = 19)

library(ggplot2)

qplot(B1, B2, data = dfAll, color = as.factor(class))

qplot(B1, B2, data = dfAll, color = as.factor(class), size = 1/class, shape = as.factor(class))

qplot(B2, B3, data = dfAll, color = as.factor(class), size = 1/class, shape = as.factor(class))

qplot(B1, B3, data = dfAll, color = as.factor(class), size = 1/class, shape = as.factor(class))

# partition data in training/testing/validation datasets

# Create validation dataset

set.seed(seed)

inBuild <- createDataPartition(y = dfAll$class, p = 0.8, list = FALSE)

validation <- dfAll[-inBuild,]

buildData <- dfAll[inBuild,]

# Create training and testing datasets

set.seed(seed)

inTrain <- createDataPartition(y = buildData$class, p = 0.75, list = FALSE)

training <- buildData[inTrain,]

testing <- buildData[-inTrain,]

# sample training dataset if too large

set.seed(seed)

nsamples <- 10000

sdfAll <- training[sample(1:nrow(training), nsamples), ]

# train the RF model

set.seed(seed)

modFit_rf <- train(as.factor(class) ~ B1 + B2 + B3 + ndvi1, method = "rf", data = sdfAll, ntree = 300)

system.time(modFit_rf <- train(as.factor(class) ~ B1 + B2 + B3, method = "rf", data = sdfAll, ntree = 200))

system.time(modFit_rf <- train(as.factor(class) ~ B1 + B2 + B3, method = "rf", data = sdfAll, ntree = 200, importance = TRUE))

# modFit_rf <- train(as.factor(class) ~ ., method = "rf", data = sdfAll) # '.' means use all predictors

# 1000 samples 100 trees 5 sec - OOB error 44.2%

# 1000 samples 500 trees 20 sec - OOB error 42.6%

# 10000 samples 200 trees 90 sec - OOB error 42%

# see Rodriguez-Galiano et al 2012 An assessment of the effectiveness of a random forest classifier for land-cover classification

# variable importance

varImp(modFit_rf)

# save trained model

save(modFit_rf, file = ".RData")

# model evaluation: confusion matrix with training data - OOB error

modFit_rf$finalModel

# model evaluation: testing

predicted_testing <- predict(modFit_rf, testing)

confusionMatrix(predicted_testing, as.factor(testing$class))

# ?confusionMatrix

# Positive Predictive Value -> user's accuracy = 1 - commission error

# Sensitivity -> producer's accuracy = 1 - omission error : accuracy by category

# model evaluation: validation

predicted_validation <- predict(modFit_rf, validation)

confusionMatrix(predicted_validation, as.factor(validation$class))

# compute quantity and allocation metrics

library(diffeR)

confMat_validation

<- unclass(confusionMatrix(predicted_validation, as.factor(validation$class))$table)

confMat_validation_pct <- confMat_validation/sum(confMat_validation) * 100

diffTablej(confMat_validation_pct, digits = 1)

overallComponentsPlot(ctmatrix = confMat_validation_pct)

categoryComponentsPlot(ctmatrix = confMat_validation_pct)

categorySourcesPlot(ctmatrix = confMat_validation_pct)

# image classification

beginCluster()

preds_rf <- clusterR(img, raster::predict, args = list(model = modFit_rf))

endCluster()

# plot classification

plot(preds_rf)

# export classification

writeRaster(preds_rf, filename = "data/processed/classification_0.tif")
