# Supplementary material for: Land use and land cover changes along the China-Myanmar Oil and Gas pipelines – Monitoring infrastructure development in remote conflict-prone regions
Source: PLoS One. 2020 Aug 19;15(8):e0237806. doi: 10.1371/journal.pone.0237806 (PMC7437919; doi:10.1371/journal.pone.0237806)
Supplement: Attachment — Submitted filename: Response to Reviewers.docx [file pone.0237806.s004.docx]

Thank you for giving us the opportunity to submit a revision of our manuscript. We truly appreciate all the constructive comments and suggestions from the reviewers.

We have adopted all the suggestions and revised each sections of our manuscript. Following the suggestion, we have followed (Olofsson et al., 2014)’s method for accuracy assessment. The following are our point-to-point responses to the reviewers’ comments.

1. I think there is still room to improve the manuscript, particularly in adhering to the good practice recommendations outlined by Olofsson et al (2014) for producing scientifically rigorous and transparent estimates of accuracy and area in land use/cover change studies. For instance, the sampling design used for the study is still unclear (e.g., did you adopt a simple random sampling, or stratified random sampling, or another sampling approach for the accuracy assessment?).

Response: Page 9, line 269-273/ Page 12&13, line 360-370_We have included the method for sampling design sued in the study.

2. What target accuracy (or accuracies) were you aiming for in the land cover maps based on your study objectives? Based on your chosen sampling design, what was the required sample size?

Response: Page 13, line 367_We reported targeted accuracy and required sample size here.

3. How many training and testing samples were used?

Response: Page 9, line 269-273_We now included the method and number of training and testing samples used in the study.

4. For the results of the accuracy assessments, you’ve responded that you have reported the accuracies (overall, user’s, and producer’s) with confidence intervals, but I do not see these reflected in Tables 1 and 2 (in commission/omission errors) or in L 357-377 in Accuracy Assessment section of Results.

Response: Page 12, Line 361: The Confidence Interval (CI) for both years is 0.95.

5. Also, the error matrices should be presented in terms of both sample counts and estimated area proportions. Currently, Tables 1 and 2 present the error matrices for land cover maps in 2010 and 2012 in terms of sample counts, but I do not see the error matrices presented yet in terms of estimated area proportions.

Response: Page 14&15. Line 395-403_we reported both sample counts and estimated area proportion.

6. Finally, Table 3 should then present the adjusted area estimates based on the results of the accuracy assessment.

Response: Adjusted area estimates are now included in table 3-6.
